# The Role of Progesterone in Elf5 Activation and Milk Component Synthesis for Cell-Cultured Milk Production in MAC-T Cells

**DOI:** 10.3390/ani14040642

**Published:** 2024-02-17

**Authors:** Hyuk Cheol Kwon, Hyun Su Jung, Do Hyun Kim, Jong Hyeon Han, Sung Gu Han

**Affiliations:** Department of Food Science and Biotechnology of Animal Resources, Konkuk University, Seoul 05029, Republic of Korea; kwonhc@konkuk.ac.kr (H.C.K.); jehceh@konkuk.ac.kr (H.S.J.); secret311@konkuk.ac.kr (D.H.K.); hyeon4970@konkuk.ac.kr (J.H.H.)

**Keywords:** progesterone, prolactin, cell-cultured milk, milk component, E74-like factor 5, MAC-T cells

## Abstract

**Simple Summary:**

Prolactin is an essential hormone to induce milk component production through secretory differentiation and activation in bovine mammary epithelial cells. Our data demonstrated that progesterone also induces the activation of E74-like factor 5 and milk component synthesis in a bovine mammary epithelial cell line, MAC-T. The utilization of progesterone (0.05 USD/mg) in an in vitro cell culture is expected to reduce the cost associated with prolactin (1250 USD/mg). Therefore, the present study suggests that progesterone can be an effective substitute for prolactin for cell-cultured milk production in cell culture models.

**Abstract:**

Prolactin is essential for mammary gland development and lactation. Progesterone also induces ductal branching and alveolar formation via initial secretory differentiation within the mammary gland. Herein, we aimed to evaluate the role of progesterone as a prolactin substitute for the production of cell-cultured milk components in MAC-T cells. Cells were treated with various hormones such as prolactin (PRL), progesterone (P4), 17β-estradiol (E2), cortisol (COR), and insulin (INS) for 5 d. MAC-T cells cultured in a P4 differentiation media (2500 ng/mL of P4, 25 ng/mL of E2, 25 ng/mL of COR, and 25 ng/mL of INS) showed similar levels of E74-like factor 5 (Elf5) and milk component synthesis (*α*-casein, *β*-casein, *α*-lactalbumin, β-lactoglobulin, and triglycerides) compared to those cultured in a PRL differentiation media (5000 ng/mL of PRL, 500 ng/mL of CORT, and 50 ng/mL of INS). The levels of *α*-casein and triglycerides in the optimal P4 differentiation media were present at comparable levels to those in the PRL differentiation media. Our results demonstrated that P4 induces the activation of Elf5 and the synthesis of milk components in MAC-T cells, similar to PRL. Therefore, P4 may be used as an effective substitute of PRL for cell-cultured milk production in in vitro frameworks.

## 1. Introduction

Milk components, such as casein, whey protein, and triglycerides, are primarily synthesized and secreted by bovine mammary epithelial cells (BMECs) of the mammary gland under autocrine and paracrine hormonal regulation [1,2]. Reproductive and metabolic hormones, such as prolactin (PRL), progesterone (P4), 17β-estradiol (E2), cortisol (CORT), insulin (INS), and growth hormone are closely related to mammary gland development, lactation, and involution [3]. Therefore, hormonal regulation is essential for milk production in the BMECs of the mammary gland.

PRL, which is the principal lactation hormone, plays an essential role in the production and maintenance of milk by stimulating the lobuloalveolar formation (secretory differentiation) and milk component synthesis (secretory activation) of BMECs [3,4]. Previous studies have mainly utilized PRL to promote the production of milk proteins and fats via BMEC differentiation [5,6,7]. However, reproductive and metabolic hormones act selectively at each stage of mammary gland development and lactation [3]. Furthermore, the application of PRL to BMECs for the production of cell-cultured milk components is limited due to the prohibitive cost of recombinant PRL [8,9].

PRL has been reported to directly induce the expression of E74-like factor 5 (Elf5) and increase the phosphorylation of signal transducer and activator of transcription 5 (STAT5) for the production of milk components during lactation [10]. In addition, previous studies have shown that forced Elf5 expression stimulates milk protein production in the murine mammary gland, whereas Elf5 deficiency impairs lobuloalveolar development during pregnancy in mice [10,11]. Taken together, Elf5 is an essential transcription factor for milk component production in the differentiation of BMECs [12].

Ovarian hormones, including P4 and E2, induce initial secretory differentiation through stimulating cell proliferation, ductal branching, and alveolar formation in BMECs during pregnancy [13]. Thereafter, the withdrawal of P4 triggers the closure of tight junctions for milk secretion during the transition from pregnancy to lactation [14]. However, previous studies have demonstrated that P4 and E2 play pivotal roles as regulators of STAT5 activation in the murine mammary gland, akin to PRL [15]. Further, P4 induced the secretory differentiation of mammary epithelial cells (MECs) through the receptor activator of nuclear factor *κ*B ligand (RANKL)-related induction of Elf5, which is essential for PRL-related secretory activation during lactation [16,17,18].

Considering that PRL-mediated regulation is required to synthesize milk components in MECs, PRL has been mainly utilized to induce secretory differentiation and activation. However, recent studies showed that P4 also induces ductal branching and alveolar formation through STAT5 and Elf5, which are the essential signaling pathways for milk production [13,15,16,17,18]. Based on these previous studies, we hypothesized that P4 could also stimulate the synthesis of milk components with the upregulation of the Elf5 signaling pathway in MAC-T cells. Therefore, the aim of this study was to investigate the role of P4 on the synthesis of cell-cultured milk components and related signaling mechanisms in MAC-T cells.

## 2. Materials and Methods

### 2.1. Chemical and Reagents

Hormones (P4, E2, INS, and CORT) were obtained from Sigma-Aldrich (St. Louis, MO, USA). PRL was purchased from ProSpec-Tany TechnoGene Ltd. (Ness-Ziona, Israel). High-glucose Dulbecco’s modified Eagle’s medium/nutrient mixture F12 (DMEM/F12) and phosphate-buffered saline (PBS) were obtained from Gibco (Grand Island, NY, USA). Fetal bovine serum (FBS), penicillin/streptomycin (P/S), and trypsin-ethylenediaminetetraacetic acid (EDTA) solution were supplied by WELGENE Inc. (Gyeongsan, Daegu, Republic of Korea).

### 2.2. Cell Culture and Treatments

MAC-T cells were cultured as described previously [19]. In brief, MAC-T cells were cultured in a T-75 cell culture flask with a high-glucose DMEM/F12 medium containing 10% FBS and 1% P/S at 37 °C in a 5% CO_2_ incubator. The MAC-T cells were sub-cultured at a density of 0.6 × 10^6^ cells in a T-75 cell culture flask using a trypsin/EDTA solution and were seeded at a density of 0.09 and 0.03 × 10^6^ cells per well into 6-well and 12-well cell culture plates, respectively. The cells were cultured to 80% confluence and treated for 5 d with various concentrations of 10–5000 ng/mL for PRL, 50–10,000 ng/mL for P4, 2.5–2500 ng/mL for E2, 2.5–2500 ng/mL for CORT, and 2.5–2500 ng/mL for INS. The control media (CM) consisted of Dulbecco’s modified Eagle’s medium/nutrient mixture F12, 10% fetal bovine serum, and 1% penicillin/streptomycin without the addition of exogenous hormones. The concentration ratio of PRL/CORT/INS for the PRL differentiation media was 100:10:1. In addition, the initial and optimal ratios of P4/E2/CORT/INS for the P4 differentiation media were 100:10:10:1 and 100:1:1:1, respectively. In addition, CORT and INS were supplemented in the PRL and P4 differentiation media for maintaining the production of stable milk components. E2 was added to the P4 differentiation media for upregulating the expression of P4 receptors in the MAC-T cells. All culture media and differentiation media were replaced every 2 d.

### 2.3. Protein Extraction and Western Blot

The cellular protein samples were prepared using a radioimmunoprecipitation assay buffer (Elpis Biotech, Daejeon, Republic of Korea), containing a protease inhibitor cocktail (Abbkine Inc., Wuhan, China). Cell lysates were centrifuged at 17,000× *g* at 4 °C for 20 min. The protein concentration in the cell supernatant was quantified using a bicinchoninic acid protein assay kit (Thermo Fisher Scientific, Rockford, IL, USA). Subsequently, protein samples were separated via sodium dodecyl sulfate–polyacrylamide gel electrophoresis and transferred onto 0.45 µm nitrocellulose membranes (GE Healthcare Biosciences, Chicago, IL, USA) using a Semi-Dry Electrophoretic Transfer Cell (Bio-Rad Laboratories, Hercules, CA, USA) and a transfer buffer (25 mM of Tris base, 192 mM of glycine, and 20% methanol, pH 8.3). The membranes were blocked using 5% bovine serum albumin in Tris-buffered saline with Tween 20 buffer (TBST) at 25 °C for 90 min. After washing three times with TBST for 15 min, the membranes were incubated with primary antibodies, including anti-Elf5 (1:1000, MBS820369, MyBioSource, San Diego, CA, USA), anti-*α*S1-casein (1:2000, SAB1401093, Sigma-Aldrich, St. Louis, MO, USA), anti-*β*-casein (1:1000, 251309, ABBIOTEC, Escondido, CA, USA), anti-*α*-lactalbumin (1:1000, MBS8501032, MyBioSource, San Diego, CA, USA), anti-*β*-lactoglobulin (1:1000, LS-C210693, LSBio, WA, USA), anti-sterol regulatory element binding protein-1 (SREBP-1, 1:1000, sc-365513, Santa Cruz Biotechnology Inc., Dallas, TX, USA), anti-peroxisome proliferator-activated receptor γ (PPARγ, 1:1000, CST#2435, Cell Signaling Technology, Danvers, MA, USA), anti-*α*-tubulin (1:5000, CST#2144, Cell Signaling Technology), and anti-glyceraldehyde 3-phosphate dehydrogenase (GAPDH, 1:20,000, ABS16, Merck Millipore, Darmstadt, Germany), at 4 °C for 15 h. Thereafter, the membranes were washed three times with TBST for 15 min and incubated with goat anti-mouse immunoglobulin G (IgG) H&L conjugated to horseradish peroxidase (1:5000, ab205719, Abcam, Cambridge, UK) and a goat anti-rabbit IgG conjugated to horseradish peroxidase (1:5000, ADI-SAB-300, Enzo Life Sciences, Lausen, Switzerland) at 25 °C for 90 min. The membranes were then washed three times with TBST for 15 min, and enhanced chemiluminescence detection reagents (Thermo Fisher Scientific) were used to visualize the protein bands. The band intensities were quantified using the Image J software, version 1.53 (National Institutes of Health, Bethesda, MD, USA).

### 2.4. RNA Extraction and Real-Time Polymerase Chain Reaction (RT-PCR)

Total RNA was obtained from the MAC-T cells using the TRIzol reagent (Ambion, TX, USA). Subsequently, cDNA was synthesized using the TOPscript RT DryMIX kit (Enzynomics, Daejeon, Republic of Korea). The gene expression levels of milk proteins (CSN1S1, CSN1S2, CSN2, CSN3, ALA, and BLG) were evaluated using the 2× real-time polymerase chain reaction (RT-PCR) Smart mix (BIOFACT Co., Ltd., Daejeon, Republic of Korea) and an RT-PCR system (Roche LightCycler^®^ 96 System, Basel, Switzerland). The thermal conditions for the PCR reaction included 60 cycles at 95 °C for 10 s, annealing at 60 °C for 10 s, and extension at 72 °C for 10 s. The mRNA expression levels were quantified using the 2^−ΔΔCt^ method, and normalization was performed using GAPDH as an internal control. The primer sequences were designed using the AmplifX software, version 1.7.0 (Nicolas Jullien, CNRS, Aix-Marseille University) (Table 1).

### 2.5. Immunofluorescence

Milk proteins (*α*S1-casein and *β*-casein) were detected using immunofluorescence staining. The MAC-T cells were fixed with 4% paraformaldehyde in PBS for 15 min. After washing three times with 0.1% Tween 20 in PBS, 0.1% Triton X-100 in PBS was used for the permeation of cells for 10 min. Subsequently, the cells were blocked with 3% bovine serum albumin at 25 °C for 90 min and incubated with anti-*α*S1-casein (1:500, SAB1401093, Sigma-Aldrich, St. Louis, MO, USA) and anti-*β*-casein (1:500, 251309, ABBIOTEC, Escondido, CA, USA) at 4 °C for 15 h. The cells were washed three times with 0.1% Tween 20 in PBS and then incubated with DyLightTM 488 conjugated-donkey anti-rabbit IgG H&L (1:1000, A120-108D2, Bethyl Laboratories, Montgomery, TX, USA) and donkey anti-rabbit IgG H&L Alexa Fluor^®^ 647 (1:1000, ab150075, Abcam, Cambridge, UK) at 25 °C for 1.5 h. After the fixation of the nuclei with 4% paraformaldehyde for 10 min, they were stained with 4,6-diamidino-2-phenylindole dihydrochloride (DAPI; 1 µg/mL) for 10 min and washed three times with 0.1% Tween 20 in PBS. The cells were captured and imaged using a Nikon Eclipse Ti2-U and a Nikon Eclipse Ts2R camera (Nikon Co., Ltd., Tokyo, Japan). The fluorescence intensities were quantified using the Image J software, version 1.53.

### 2.6. Oil Red O Staining

Intracellular triglycerides, a major component of milk fat, were determined using oil red O dye (Sigma-Aldrich). The MAC-T cells were fixed using 10% formalin at 25 °C for 1 h and washed with 60% isopropanol. After completely drying, the oil red O working solution was used to stain the cells for 10 min. The unbound dye was removed using deionized distilled water, whereafter the stained cells were captured and imaged using a Nikon Eclipse Ti2-U and a Nikon Eclipse Ts2R camera. The oil red O staining areas were quantified using the Image J software, version 1.53.

### 2.7. Enzyme-Linked Immunosorbent Assay (ELISA), Triglyceride Assay, and Freeze Drying

The levels of the milk components *α*-casein and triglycerides in the culture media were determined using the Bovine Casein Alpha (CSN1) ELISA Kit (Bioss Antibodies, Woburn, MA, USA) and a Triglyceride Assay Kit (Abcam, Cambridge, UK), as per the manufacturer’s instructions. The culture media were collected in 1.7 mL micro tubes after the differentiation of the MAC-T cells for 5 d and centrifuged at 17,000× *g* and 4 °C for 20 min. The supernatants of the culture media were preserved at −80 °C until use. In addition, optimal P4 differentiation media samples of 10 mL were lyophilized using a Virtis freeze dryer (Virtis Co. Inc., Gardner, NY, USA).

### 2.8. Statistical Analysis

All experiments were performed at least three times independently. The experimental data are represented as the mean ± the standard error of the mean (SEM) and were analyzed using the SPSS-PASW statistics software, version 22.0 (SPSS Inc., Chicago, IL, USA). A two-tailed Student’s *t*-test was used for two-group comparisons. A one-way analysis of variance with the post hoc Dunnett test and Duncan’s new multiple range test were used for three-group and multiple comparisons, respectively. Statistical significance was set at *p* < 0.05.

## 3. Results

### 3.1. Selection of PRL and P4 Concentrations Based on Cell Differentiation Markers

**Figure 1:**
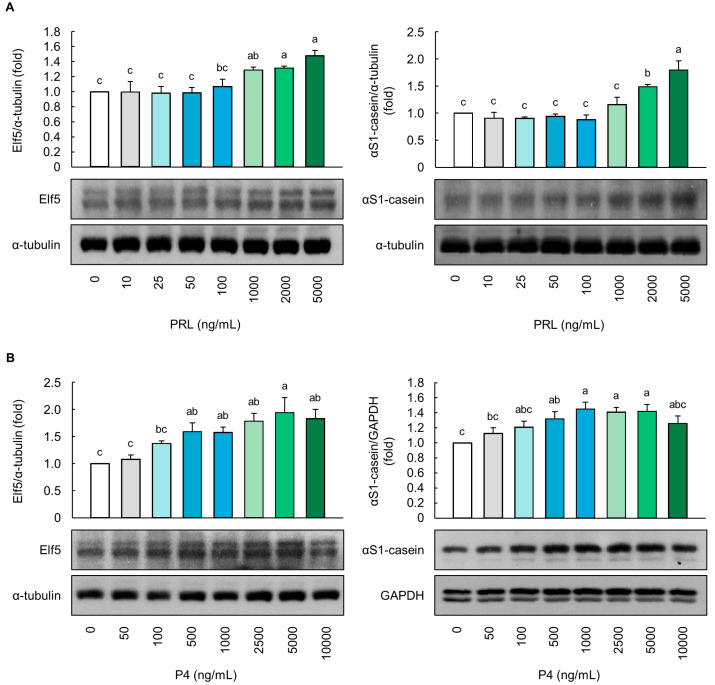
Evaluation of alveolar differentiation based on milk protein-related markers in MAC-T cells. Protein levels of E74-like factor 5 (Elf5) and *α*S1-casein in (**A**) prolactin (PRL)- and (**B**) progesterone (P4)-treated MAC-T cells for 5 d (*n* = 3). The ratio of PRL/cortisol (CORT)/insulin (INS) in PRL differentiation media was 100:10:1, and the ratio of P4/17β-estradiol (E2)/CORT/INS in P4 differentiation media was 100:10:10:1. Glyceraldehyde-3-phosphate dehydrogenase (GAPDH) and *α*-tubulin were used as loading controls. Representative images were selected from three independent replicates. Data are expressed as mean ± standard error of the mean. Different superscript letters indicate significant differences (*p* < 0.05).

At a concentration of up to 1000 ng/mL, PRL did not affect the protein levels of Elf5 and *α*S1-casein in the MAC-T cells (*p* > 0.05, Figure 1A). However, cells treated with 2000 and 5000 ng/mL of PRL showed significantly higher protein levels of both Elf5 and *α*S1-casein compared to the control cells, with the highest levels observed with 5000 ng/mL of PRL (*p* < 0.05, Figure 1A). In addition, the P4 (500–5000 ng/mL) treatment significantly upregulated the Elf5 and *α*S1-casein protein levels in a dose-dependent manner (*p* < 0.05, Figure 1B). However, no significant increase was noted at 10,000 ng/mL, compared to 5000 ng/mL of P4 (*p* > 0.05).

### 3.2. Optimization of P4 Concentration Based on Cell Differentiation Markers

**Figure 2:**
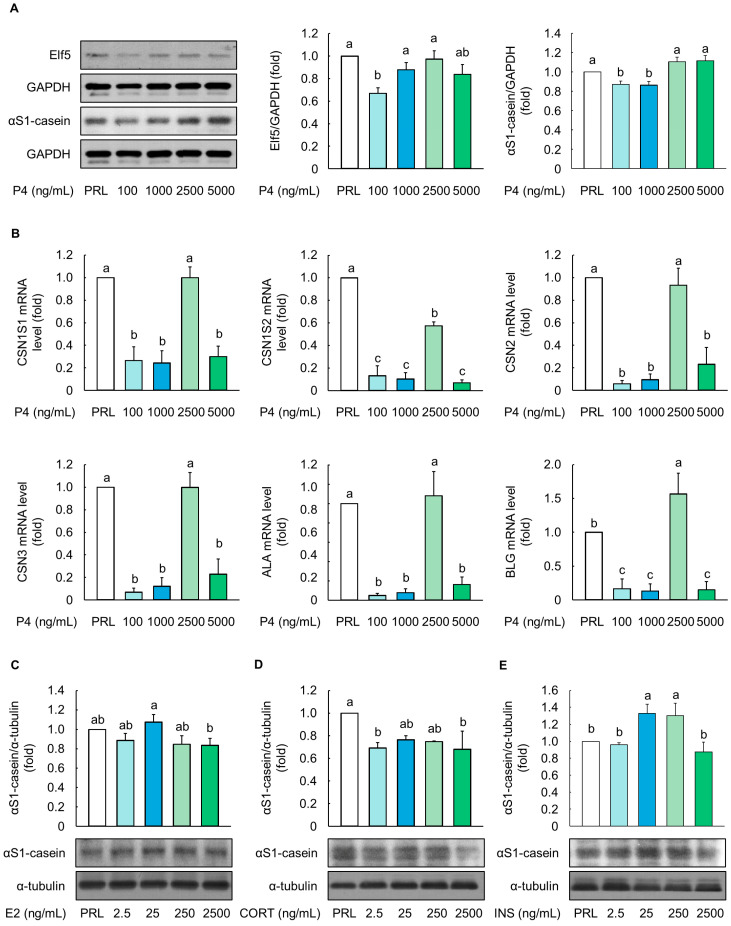
Optimization of progesterone concentrations based on milk protein-related markers in MAC-T cells. (**A**) Protein expression levels (E74-like factor 5 (Elf5) and *α*S1-casein) and (**B**) mRNA levels (CSN1S1, CSN1S2, CSN2, CSN3, *α*-lactalbumin (ALA) and β-Lactoglobulin (BLG)) in MAC-T cells treated with 5000 ng/mL prolactin (PRL) and 100–5000 ng/mL progesterone (P4) for 5 d (*n* = 3). The ratio of PRL/cortisol (CORT)/insulin (INS) in PRL differentiation media was 100:10:1, and the ratio of P4/17β-estradiol (E2)/CORT/INS in P4 differentiation media was 100:10:10:1. Protein level of *α*S1-casein in MAC-T cells treated with (**C**) E2, (**D**) CORT, and (**E**) INS at various concentrations (2.5–2500 ng/mL) (*n* = 3). Glyceraldehyde-3-phosphate dehydrogenase (GAPDH) and *α*-tubulin were used as loading controls. Representative images were selected from three independent replicates. Data are expressed as mean ± standard error of the mean. Different superscript letters indicate significant differences (*p* < 0.05).

In the subsequent experiments, four different concentrations of P4 (100, 1000, 2500, and 5000 ng/mL) were evaluated to determine their optimal concentration in culture media. PRL at 5000 ng/mL was used as a positive control. The cells treated with 1000, 2500, and 5000 ng/mL of P4 had comparable protein levels of Elf5 compared to those of the positive control (*p* > 0.05, Figure 2A). P4 (2500 and 5000 ng/mL) similarly upregulated the protein level of *α*S1-casein. Furthermore, the mRNA expression levels of milk protein-related genes such as CSN1S1, CSN1S2, CSN2, CSN3, ALA, and BLG were similar between cells treated with 2500 ng/mL of P4 and the positive control (*p* > 0.05, Figure 2B). Therefore, 2500 ng/mL was selected as the optimal concentration of P4 for the differentiation media. Furthermore, the optimal E2, CORT, and INS concentrations were determined to be 25 ng/mL, based on the levels of *α*S1-casein protein when compared with those under PRL treatment (Figure 2C–E).

### 3.3. Effects of Optimal P4 Differentiation Media on Milk Protein Production

**Figure 3:**
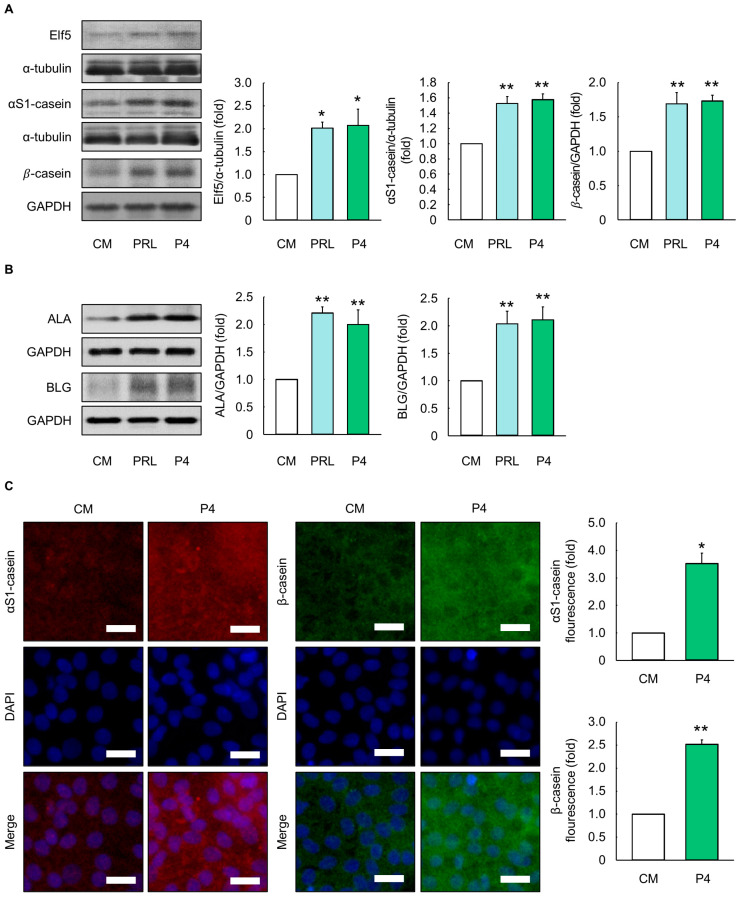
Evaluation of milk protein production in MAC-T cells cultured with optimal progesterone differentiation media. (**A**) Protein expression levels of E74-like factor 5 (Elf5), *α*S1-casein, β-casein, (**B**) *α*-lactalbumin (ALA), and β-Lactoglobulin (BLG) in MAC-T cells treated with prolactin (PRL) and progesterone (P4) differentiation media for 5 d (*n* = 3). (**C**) mCherry, green fluorescence immunofluorescence (GFP), and 4,6-diamidino-2-phenylindole dihydrochloride (DAPI) images of *α*S1-casein (red) and β-casein (green) in MAC-T cells treated with optimal P4 differentiation media for 5 d (*n* = 3). Control media (CM) consisted of Dulbecco’s modified Eagle’s medium/nutrient mixture F12, 10% fetal bovine serum, and 1% penicillin/streptomycin without addition of exogenous hormones. The concentrations of PRL, cortisol (CORT), and insulin (INS) in PRL differentiation media were 5000, 500, and 50 ng/mL, respectively, whereas the concentrations of P4, 17β-estradiol (E2), CORT, and INS in optimal P4 differentiation media were 2500, 25, 25, and 25 ng/mL, respectively. Glyceraldehyde-3-phosphate dehydrogenase (GAPDH) and *α*-tubulin were used as loading controls. The magnification of images is 200×. Scale bar indicates 25 µm. Representative images were selected from three independent replicates. Data are expressed as mean ± standard error of the mean. * (*p* < 0.05) and ** (*p* < 0.01) show a significant difference compared to CM.

MAC-T cells were cultured to evaluate milk protein production using PRL differentiation media (5000 ng/mL of PRL; 500 ng/mL of CORT; and 50 ng/mL of INS) and optimal P4 differentiation media (2500 ng/mL of P4; 25 ng/mL of E2; 25 ng/mL of CORT; and 25 ng/mL of INS). Cells in the optimal P4 differentiation media had similar Elf5 and milk protein (*α*S1-casein, *β*-casein, *α*-lactalbumin (ALA) and *β*-lactoglobulin (BLG)) levels relative to those cultured in PRL differentiation media (*p* > 0.05, Figure 3A,B). Furthermore, there was a significant difference in protein expression levels in cells between the CM and optimal P4 differentiation media (*p* < 0.05, Figure 3A,B). The latter also exhibited enhanced fluorescence signals of *α*S1-casein and *β*-casein when compared to the CM (*p* < 0.05, Figure 3C).

### 3.4. Effects of Optimal P4 Differentiation Media on Milk Fat Production

**Figure 4:**
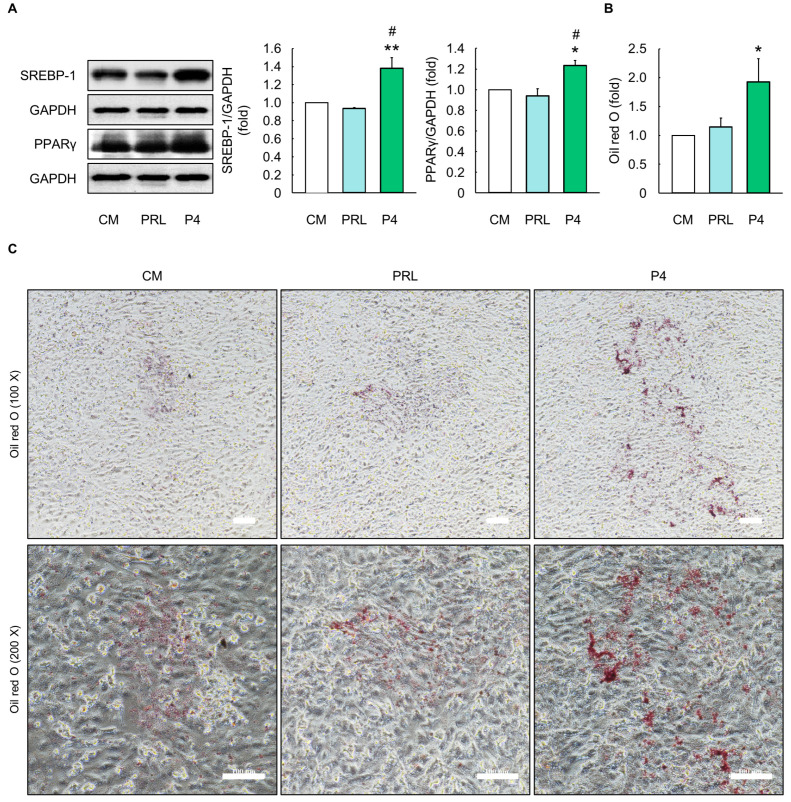
Evaluation of milk fat production in MAC-T cells cultured with optimal progesterone differentiation media. (**A**) Protein levels of sterol regulatory element binding protein-1 (SREBP-1) and peroxisome proliferator-activated receptor γ (PPAR γ) in MAC-T cells treated with prolactin (PRL) and progesterone (P4) differentiation media for 5 d (*n* = 3). (**B**) Quantification of areas stained with oil red O and (**C**) microscopy images in MAC-T cells treated with PRL and optimal P4 differentiation media for 5 d (*n* = 3). Control media (CM) consisted of Dulbecco’s modified Eagle’s medium/nutrient mixture F12, 10% fetal bovine serum, and 1% penicillin/streptomycin without addition of exogenous hormones. The concentrations of PRL, cortisol (CORT), and insulin (INS) in PRL differentiation media were 5000, 500, and 50 ng/mL, respectively, and concentrations of P4, 17β-estradiol (E2), CORT, and INS in optimal P4 differentiation media were 2500, 25, 25, and 25 ng/mL, respectively. Glyceraldehyde-3-phosphate dehydrogenase (GAPDH) was used as a loading control. The magnification of images is 100× and 200×. Scale bar indicates 100 µm. Representative images were selected from three independent replicates. Data are expressed as mean ± standard error of the mean. * (*p* < 0.05) and ** (*p* < 0.01) show a significant difference compared to CM. # (*p* < 0.05) shows a significant difference compared to the PRL differentiation media-treated cells.

The optimal P4 differentiation media significantly increased the protein levels of SREBP-1 and PPARγ in the MAC-T cells compared to those in the CM (*p* < 0.05, Figure 4A). Furthermore, cells cultured in the optimal P4 differentiation media had more oil red O-stained areas than those cultured in the CM (*p* < 0.05, Figure 4B,C).

### 3.5. Effects of Optimal P4 Differentiation Media on Milk Component Production

**Figure 5:**
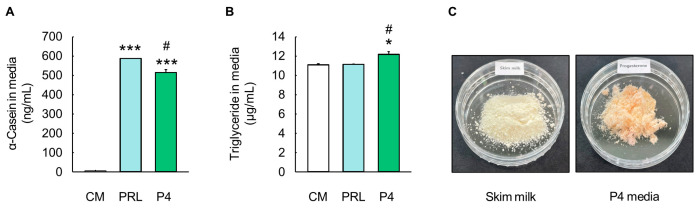
Evaluation of milk component production by MAC-T cells cultured in optimal progesterone differentiation media. (**A**) Milk protein *α*-casein levels in prolactin (PRL) and progesterone (P4) differentiation media (*n* = 3). (**B**) Milk triglyceride levels in PRL and optimal P4 differentiation media (*n* = 3). (**C**) Skim milk and lyophilized optimal P4 differentiation media. Control media (CM) consisted of Dulbecco’s modified Eagle’s medium/nutrient mixture F12, 10% fetal bovine serum, and 1% penicillin/streptomycin without addition of exogenous hormones. The concentrations of PRL, cortisol (CORT), and insulin (INS) in PRL differentiation media were 5000, 500, and 50 ng/mL, respectively, whereas concentrations of P4, 17β-estradiol (E2), CORT, and INS in optimal P4 differentiation media were 2500, 25, 25, and 25 ng/mL, respectively. Data are expressed as mean ± standard error of the mean. * (*p* < 0.05) and *** (*p* < 0.001) show a significant difference compared to CM. # (*p* < 0.05) shows a significant difference compared to the PRL differentiation media-treated cells.

The levels of *α*-casein and triglycerides in the culture media were analyzed to evaluate milk protein and fat production (Figure 5). The optimal P4 differentiation media showed significantly higher levels of *α*-casein (515.21 ± 14.89 ng/mL) compared to the CM (5.33 ± 0.08 ng/mL) (*p* < 0.001, Figure 5A), and the *α*-casein levels in the optimal P4 differentiation media were similar to those in the PRL differentiation media (588.40 ± 0.12 ng/mL) (Figure 5A). In addition, the triglyceride contents were significantly higher in the optimal P4 differentiation media (12.19 ± 0.29 µg/mL) than in the CM (11.11 ± 0.11 µg/mL) (*p* < 0.05, Figure 5B). In particular, the level of triglycerides in the optimal P4 differentiation media was higher than that in the PRL differentiation media (11.16 ± 0.03 µg/mL) (*p* < 0.05, Figure 5B). Moreover, 10 mL of the lyophilized optimal P4 differentiation media showed a similar appearance to skim milk and contained approximately 5 µg of *α*-casein and 120 µg of triglycerides, as determined via the quantitative analysis of milk components (Figure 5C).

## 4. Discussion

The development and lactation of mammary glands are primarily regulated under the control of reproductive and metabolic hormones [20]. In particular, PRL is essential to induce the differentiation of MECs into alveolar structures for the synthesis and secretion of milk in the process of gestation [21]. Previous studies have shown that mice lacking the PRL receptor exhibit dysfunctional ductal branching and alveolar bud formation in the mammary gland [22,23]. In addition, PRL has been reported to promote alveolar differentiation and milk production through Janus kinase 2–STAT5–Elf5 and receptor activator of nuclear factor *κ*B ligand (RANKL) signaling [24]. Various studies have applied PRL for secretory differentiation and milk production in cell culture models [25,26,27]. However, such treatment is quite expensive due to the cost of recombinant PRL [8,9]. P4 induces extensive side-branching and lobuloalveolar genesis for mammary gland development during pregnancy [28]. In previous studies, depletion of the P4 receptor and PRL in mice impaired side-branching and lobuloalveolar development [23,29,30]. Interestingly, these defects were rescued through the administration of P4, indicating that P4 may interact with PRL for the lobuloalveolar development of the mammary gland during puberty and pregnancy [23]. Furthermore, P4 was shown to induce ductal side-branching and initial secretory differentiation through the RANKL–Elf5 signaling pathway in mouse mammary glands, along with PRL [18]. In fact, a sharp decrease in P4 concentrations in the presence of PRL, CORT, and INS during the transition from pregnancy to lactation triggers the onset of milk production. Thus, the presence of P4 during lactation hinders milk secretion via the delay of tight junction closure [14,31]. However, considering that the PRL-induced signaling pathway during secretory differentiation and activation (i.e., STAT5 and Elf5) is similar to that of P4, it was assumed that P4 has the potential to synthesize milk components in cell culture conditions. Therefore, we evaluated the role of P4 in the synthesis of cell-cultured milk components in MAC-T cells.

Hormones for the alveolar development and differentiation of BMECs are supplied via the extensive vascular network of the mammary gland [32]. Therefore, the concentration range of hormones for in vitro MAC-T cell culture can be established based on their levels in the bloodstream of lactating dairy cows with high milk productivity and nutritional content. Moreover, since MAC-T cells are cultivated with culture media, which consists of a basal medium (a complement of amino acids, glucose, and vitamins), serum (source of growth factor, hormones, and attachment factors), and several supplements [33], the concentrations of both endogenous and exogenous hormones were collectively taken into account for our in vitro cell culture setting. The plasma concentration of PRL, which plays an essential role in milk synthesis and secretion, was found to reach 50 ng/mL in lactating dairy cows 3–5 d prior to the initiation of lactation [34,35]. Therefore, the criterion for the hormone concentration was aligned to that just before lactation onset, and the differentiation period was set at 5 d. According to previous reports, the following hormone concentrations were investigated: 50 ng/mL for PRL, 4.5–6.5 ng/mL for P4, 0.5–0.8 ng/mL for E2, 5 ng/mL for CORT, and 0.25–0.5 ng/mL for INS, with a ratio of 100:10:1:10:1 [34,35,36,37,38,39,40]. In addition, the hormone concentrations of exogenous PRL and P4 added to the culture media of MECs were 5000 ng/mL [7,41]. Taken together, the concentration criteria of hormones were established based on the endogenous concentration in plasma and the exogenous concentration in culture media. However, the ratio of P4 was adjusted to be consistent with that of PRL, and the ratio of CORT to INS was fixed to maintain stable milk component production [42]. Therefore, the initial ratios of PRL/CORT/INS and P4/E2/CORT/INS were determined as 100:10:1 and 100:10:10:1.

Since both PRL and P4 promote the alveolar development and differentiation of MECs through the milk production-related Elf5 signaling pathway [11,13], Elf5 and *α*S1-casein were evaluated as markers for selecting the initial concentrations of PRL and P4. At a concentration of 2500 ng/mL, P4 increased the protein levels and gene expression of Elf5 and milk proteins in MAC-T cells, to an extent similar to PRL. The hormone concentrations of E2, CORT, and INS were optimized based on a P4 concentration of 2500 ng/mL. Therefore, the optimal concentrations of P4, E2, CORT, and INS were established as 2500, 25, 25, and 25 ng/mL, with a ratio of 100:1:1:1, respectively. These results indicated that the exogenous hormones in the in vitro culture directly increased the activation of Elf5 and the synthesis of milk components more than endogenous hormones. Subsequently, alveolar differentiation and milk productivity in MAC-T cells were evaluated through a comparison of optimal P4 differentiation media with PRL differentiation media. Surprisingly, MAC-T cells cultured with the optimal P4 differentiation media synthesized milk components (*α*-casein, *β*-casein, ALA, BLG, and triglycerides) with an increase in Elf5, which was similar to those cultured with PRL. Furthermore, the optimal P4 differentiation media increased the milk fat productivity to a greater extent than the PRL differentiation media. The exogenous P4 in the culture media binds to the P4 receptor, which is a member of the nuclear receptor family of ligand-dependent transcription factors [43]. According to previous studies, the P4 receptor was expressed only in alveolar epithelial cells [44], and the expression of the P4 receptor was under the control of E2 in the mammary gland of a Holstein cow [45]. In particular, the P4 receptor, which is an E2 receptor target gene, was upregulated by E2 in bovine mammary tissues [45]. Indeed, a previous study showed that MAC-T cells inherently express the P4 receptor, and E2-treated cells increase the protein level of the P4 receptor compared to control cells [46]. Therefore, our data showed that E2 in optimal P4 differentiation media upregulates the P4 receptor of MAC-T cells, and the binding of both exogenous P4 and the P4 receptor induces the activation of Elf5 and the production of milk components.

## 5. Conclusions

This study supports that P4, at a level similar to PRL, promotes the differentiation-related Elf5 signaling pathway and milk component synthesis (*α*-casein, *β*-casein, ALA, BLG, and triglycerides) in MAC-T cells. Additionally, our in vitro data demonstrated that MAC-T cells cultured in optimal P4 differentiation media secreted *α*-casein (0.5 µg/mL) and triglycerides (12 µg/mL) into the media. To the best of our knowledge, only a few previous studies have reported milk component production in MAC-T cells using P4. Therefore, our study provides original and novel scientific knowledge about the role of P4 in the activation of Elf5 and the production of cell-cultured milk components. Our data suggest that P4 can serve as an effective hormone for the production of cell-cultured milk components in MAC-T cells. Our findings also offer fundamental insights into how P4 can modulate the synthesis of milk components in in vitro cell culture models.

## Figures and Tables

**Table 1:** Primers for real-time polymerase chain reaction analysis.

Gene ^1^	Primer Sequence 5′–3′
CSN1S1 (Bos Taurus)	(F) ACT GAG GAT CAA GCC ATG GAA G (R) GAA TGT GCT TCT GCT CAA CAC T
CSN1S2 (Bos Taurus)	(F) AAT CCA TGC CCA ACA GAA AG (R) TCA GAG CCA ATG GGA TTA GG
CSN2 (Bos Taurus)	(F) CTG GAA TTA ACT GCT TCT ACC T (R) TAC TCT GCG ATT TGT CTT ATT GA
CSN3 (Bos Taurus)	(F) GGC GAG CCT ACA AGT ACA CCT A (R) GGA CTG TGT TGA TCT CAG GTG G
ALA (Bos Taurus)	(F) CCT GAA TGG GTC TGT ACC ACG TTT (R) ATG TTG CTT GAG TGA GGG TTC TGG
BLG (Bos Taurus)	(F) AGG CCT CCT ATT GTC CTC GT (R) GCA AAG GAC ACA GGG AGA AG
GAPDH (Bos Taurus)	(F) ATG ATT CCA CCC ACG GCA AGT T (R) ATC ACC CCA CTT GAT GTT GGC A

^1^ CSN, casein; ALA, α-lactalbumin; BLG, β-lactoglobulin; GAPDH, glyceraldehyde 3-phosphate dehydrogenase.

## Data Availability

The RNA-seq data from this study were deposited in the NCBI (https://www.ncbi.nlm.nih.gov/ accessed on 1 November 2021). Data are contained within the article.
